# Distinct substrate specificity and physicochemical characterization of native human hepatic thymidine phosphorylase

**DOI:** 10.1371/journal.pone.0202826

**Published:** 2018-08-23

**Authors:** Taesung Oh, Mahmoud H. el Kouni

**Affiliations:** Department of Pharmacology and Toxicology, Comprehensive Cancer Center, Center for AIDS Research, General Clinical Research Center, The University of Alabama at Birmingham, Birmingham, AL, United States of America; Boston University Henry M Goldman School of Dental Medicine, UNITED STATES

## Abstract

Thymidine phosphorylase (TP; EC 2.4.2.4) is involved regulation of intra- or extracellular thymidine concentration, angiogenesis, cancer chemotherapy, radiotherapy, as well as tumor imaging. Although the liver is main site of pyrimidine metabolism and contains high levels of TP, nonetheless, purification and characterization of human hepatic TP has not been accomplished. We here report the purification and characterization of native human hepatic TP. The enzyme was purified to apparent homogeneity by a procedure shorter and more efficient than previously reported methods. Human hepatic TP has an apparent *K*_thymidine_ of 285 ± 55 μM. Like the enzyme from other tissues, it is highly specific to 2'-deoxyribosides. However, in contrast to TP from other normal tissues, the hepatic enzyme is active in the phosphorolysis of 5'-deoxy-5-fluorouridine, and the riboside 5-fluorouridine. Furthermore, native hepatic TP exists in different aggregates of 50 kDa subunits, with unknown aggregation factor(s) while TP from extra tissues exists as a homodimer. Isoelectric point was determined as 4.3. A total of 65 residues in the *N*-terminal were sequenced. The sequence of these 65 amino acids in hepatic TP has 100% sequence and location homology to the deduced amino acid sequence of the platelet derived-endothelial cell growth factor (PD-ECGF) cDNA. However, and contrary to PD-ECGF, the *N*-terminal of hepatic TP is blocked. The block was neither *N*-formyl nor pyrrolidone carboxylic acid moieties. The differences in substrate specificities, existence in multimers, and weak interaction with hydroxyapatite resin strongly suggest that hepatic TP is distinct from the enzyme in normal extrahepatic tissues. These results may have important clinical implications when TP is involved in activation or deactivation of chemotherapeutic agents in different tissues.

## Introduction

Thymidine phosphorylase (TP; EC 2.4.2.4) is an important enzyme of the pyrimidine salvage pathways. It catalyzes the reversible phosphorolysis of thymidine, deoxyuridine, but not deoxycytidine, and their analogues, to their respective nucleobases and 2-deoxy-α-d-ribose-1-phosphate as follows:

$$\mathrm{Pyrimidine}\;2'‑\mathrm{deoxyriboside}+P_{i}\;\Xi \;\mathrm{Pyrimidine}\;\mathrm{nucleobase}+2‑\mathrm{deoxy}‑\alpha ‑D‑\mathrm{ribose}‑1‑\mathrm{phosphate}$$

The biological significance of TP has several aspects. It has a role in maintaining the integrity of the blood vessels, and its activity is an essential step in the regulation of intra- or extracellular thymidine concentration and homeostasis in mammalian cells [1–15]. The enzyme is identical to the platelet-derived endothelial cell growth factor (PD-ECGF) [16–18] which is involved in the process of angiogenesis, neoplastic tissue growth, and metastases. In addition, mutation of the TP gene is associated with mitochondrial neurogastrointestinal encephalomyopathy (MNGIE), an autosomal recessive human disease exhibiting multiple deletions of skeletal muscle mitochondrial DNA (peripheral neuropathy, myopathy, leukoencephalopathy, lactic acidosis, gastrointestinal dysmotility, progressive external ophthalmoplegia, and thin body habitus [18]. TP, also plays a critical role in cancer chemotherapy, radiotherapy, as well as tumor imaging as it activates or deactivates some of most frequently used chemotherapeutic pyrimidine nucleoside analogues (e.g. 5-fluorouracil, 5'-deoxy-5-fluorouridine, 5-fluoro-2'-deoxyuridine, 5-iodo-2'-deoxyuridine, 5-bromo-2'-deoxyuridine, capecitabine [19–22].

The liver is the major site of pyrimidine metabolism and contains high levels of TP [20, 23–27]. Nevertheless, little is known about the substrate specificity, physiochemical characteristics, physiological role(s) and regulation of human hepatic TP. In the present study, human hepatic TP was purified to apparent homogeneity with relatively high yield (40%) using a new, shorter, and more efficient method to purify TP on a large scale than the previously reported methods. In addition the substrate specificity and physicochemical properties of the purified enzyme were determined and exhibited differences from other TPs from normal extra hepatic tissues.

## Materials and methods

### Chemicals

[2-^14^C]thymidine (56 Ci/mol), [2-^14^C]uridine (56 Ci/mol), [2-^14^C]2ʹ-deoxyuridine (56 Ci/mol), [2-^14^C]5-fluorouridine (56 Ci/mol), [2-^14^C]5-fluoro-2´-deoxyuridine (56 Ci/mol), and [6-^3^H]5´-deoxy-5-fluorouridine (7 Ci/mmol) were purchased from Moravek Biochemicals Inc., Brea, CA; Macherey Nagel Polygram Silica Gel G/UV_254_ thin layer chromatography plates from Fisher Scientific, Pittsburgh, PA.; DEAE-cellulose anion exchanger (fast flow; fibrous form), Phenyl Sepharose CL 4B, Sephadex G-150, Sephadex G-100, Polybuffer exchanger 94, Polybuffer 74, and gel filtration calibration (LMW and HMW) kit from Sigma Chemical Co., St. Louis, MO; Bio-Gel HTP hydroxyapatite, silver stain plus kit, Bio-Rad protein assay kit, Coomassie Brilliant Blue R-250, alkaline Phosphatase, 5-bromo-4-chloro-3-indoylphosphate *p*-toluidine salt, and *p*-nitro blue tetrazolium chloride from Bio-Rad Laboratories, Hercules, CA; Diaflo ultrafiltration membrane (YM30, 43 mm diameter), and Centricon-30 concentrator from Amicon Inc., Beverly, MA; Spectra/Por 2 membrane dialysis tubing (12–14,000 molecular weight cut off, 105 mm flat width) from Spectrum Medical Industries Inc., Houston, TX; Ultrafree-MC filters of 10,000 NMWL regenerated cellulose membrane from Millipore Corp., Bedford, MA; TaqStart Antibody from Clontech Laboratories Inc., Palo Alto, CA; Immobilon-P transfer membrane, and anhydrous hydrazine from Pierce Chemical Company Inc., Rockford, IL; Goat Anti-Rabbit IgG-AP (conjugated with alkaline phosphatase) from Southern Biotechnology Associates Inc., Birmingham, AL; TWEEN 20 (polyoxyethylene (20) sorbitan monolaurate), and Cyanogen bromide (CNBr) from Aldrich Chemical Company Inc., Milwaukee, WI. All other chemicals were obtained from Sigma Chemical Co., St. Louis, MO. Rabbit antisera against human placenta TP was generously provided by Dr. Edward L. Schwartz, Clinical Oncology Program, Albert Einstein Cancer Center, Bronx, NY.

### Preparation of the cytosol

Human livers and placentas were obtained from the Tissue Procurement Office at the University Alabama at Birmingham, Birmingham, AL. Livers were provided as autopsy material. IRB committee, the University of Alabama at Birmingham, approved the study and waived the requirement for informed consent. Samples of 200 g were homogenized (1:3, w/v) in Buffer A (20 mM potassium phosphate, pH 8.0; 1 mM DTT, 1 mM EDTA, 1 mM phenylmethanesulfonyl fluoride) in a Waring Blender. The homogenate was centrifuged at 50,000 x g for 1 hr and the supernatant fluids (cytosol) was collected and filtered through a funnel equipped with 3 layers of a surgical U.S.P. Type VII gauze sponges (Johnson & Johnson Products Inc., New Brunswick, NJ).

### Enzyme purification

Human liver cytosol (600 mL, ~ 24 g protein) was applied to DEAE-cellulose column (7.5 x 50 cm) pre-equilibrated with Buffer A. The column was eluted with 6 L of Buffer A, followed by a 4 L of linear gradient of NaCl (0–1.0 M) in Buffer A. Fractions of 40 mL were collected at a flow rate of 150 ml/hour. Fractions (201–245) containing TP activity (1.8 L, 5.4 g protein) were pooled and adjusted to 0.5 M ammonium sulfate and applied to Phenyl Sepharose CL 4B column (7.5 x 20 cm) pre-equilibrated with Buffer B (0.5 M ammonium sulfate in Buffer A). The column was eluted with 2 L of Buffer B, 3.5 L of 0–20% 2-ethoxyethanol gradient in Buffer A, 1.5 L of 20% 2-ethoxyethanol in Buffer A and finally with a 20–40% linear gradient of 2-ethoxyethanol in 5 L of Buffer A. Fractions of 25 mL were collected at a flow rate of 150 mL/hr. Fractions (322–346 = 625 mL), containing TP activity were pooled and dialyzed in 5% ethylene glycol monoethyl ether in 10 mM phosphate buffer (pH 8.0) then in 10 mM phosphate buffer (pH 8.0) overnight, twice. The volume of the dialyzed Phenyl Sepharose CL 4B TP fraction was reduced to 50 mL in an Amicon stir cell model 8050 (Amicon Inc., Beverly, MA) equipped with an YM 30 membrane. The dialyzed and concentrated Phenyl Sepharose TP active fraction (50 mL, 9.2 mg protein) was applied to a hydroxyapatite column (5 x 20 cm) equilibrated with Buffer C (2 mM potassium phosphate, pH 8.0; 1 mM DTT, 1 mM EDTA). The column was washed with 800 mL of Buffer C and eluted with 1 L of a linear gradient of 20–150 mM potassium phosphate buffer (pH 8.0) containing 1 mM DTT and 1 mM EDTA. Fractions of 11 mL were collected at a flow rate of 50 mL/hour. All purification steps were performed in a cold room at 4°C. Protein concentrations were determined by the Bradford [28] method using bovine γ-globulin as a standard. Polyacrylamide gel electrophoresis was used to assess the homogeneity and estimate the relative molecular mass of TP after each purification step.

### Enzyme assay

TP activity was measured radioisotopically by monitoring the formation of radioactive nucleobases from their respective nucleosides as described previously [20]. The standard assay mixture contained 20 mM potassium phosphate (pH 8.0), 1 mM DTT, 1 mM EDTA, 0.25 mM nucleoside (2..24 Ci/mol), unless otherwise specified, and 25 uL of enzyme in a final volume of 50 uL. The incubation was carried out for 20 min at 37°C. Under these conditions the activity was linear with time and enzyme concentration. The reactions were initiated by addition of enzyme and terminated by boiling in a water bath for 2 min followed by freezing for at least 20 min. Proteins were removed by centrifugation and 10 uL of the supernatant fluid were spotted on silica gel thin layer chromatography plates. The plates were then developed in a mixture of chloroform:methanol:acetic acid (90:5:5, v/v/v). The *R*_*f*_ values were: thymidine, 0.14; uridine, 0.07; deoxyuridine, 0.1; 5-fluorouridine, 0.05; 5-fluoro-2'-deoxyuridine, 0.07; 5-fluoro-5'-deoxyuridine, 0.16; thymine, 0.62; uracil, 0.43; and 5-fluorouracil, 0.41. The amount of radioactivity in the substrate and product were detected and quantified on a percentage basis using a Berthold LB-2821 Automatic TLC Linear Analyzer. Apparent *K*_m_ values were calculated using a computer program, which employs the Wilkinson-Cleland procedure as previously described [20, 25].

### Polyacrylamide gel electrophoresis

Analytical SDS-PAGE was performed as follows, samples were combined with the sample buffer containing 63 mM Tris-Cl (pH 6.8), 2% SDS, 10% glycerol, 0.03% bromophenol blue and 5% β-mercaptoethanol and placed in a boiling water bath for 2 min before being loaded onto a 10% polyacrylamide gel slab containing 0.1% SDS [29]. Electrophoresis was carried out at 100 constant voltage. Analytical native-PAGE was carried out under the same conditions, but without SDS or β-mercaptoethanol and the samples were not boiled. Gels were stained for protein using either Coomassie Blue R-250 or Silver Stain Plus kit [30].

Preparative native-PAGE was performed under the same conditions described above for the analytical native-PAGE electrophoresis. The preparative electrophoresis was performed with an on-line electrophoresis system on a Bio-Rad 491 Prep Cell (Bio-Rad Laboratories, Hercules, CA) using a rod type gel rather than a slab gel. The gel solution was poured into the gel tube to a vertical height of 4.6 cm. After a pre-run for 1 hr, 5 mL of sample (0.2 g protein) was loaded over the top of the gel and electrophoresis was performed at a constant voltage of 300 V at 4°C. Proteins were eluted during the electrophoresis from the bottom of the gel and 10.5 mL fractions were collected at 31 mL/hr flow rate. TP activity was determined in each fraction by the standard assay.

### Immunoblotting

Samples of TP were subjected to native-PAGE. Separated proteins in the gel were electrotransfered to a nitrocellulose membrane using a Mini Trans-Blot^®^ Electrophoretic Transfer Cell (Bio-Rad Laboratories, Hercules, CA) in a horizontal sandwich configuration at a constant voltage of 30 V overnight. After the electrotransfer, the membrane was incubated for 1 hr with gentle agitation in borate saline solution (100 mM boric acid, pH 8.45; 25 mM sodium borate, 75 mM sodium chloride) containing 3% BSA, further incubated for 2 hr in borate saline solution containing 1% BSA, 0.2% TWEEN 20 and TP antisera (1:2000 dilution) and then washed for 10 min in borate saline solution containing 0.2% TWEEN 20. The wash was repeated twice. The membrane was then incubated for 1 hr in borate saline solution containing 3% BSA, alkaline phosphatase-conjugated goat anti-rabbit IgG (1:5000 dilution) and 0.2% TWEEN 20, washed three times for 5 min each in borate saline solution containing 0.2% TWEEN 20. After the washes, the immunoblot was developed for about 20 min in color development solution (100 mM NaHCO_3_, pH 9.5; 0.5 mM MgCl_2_, 0.5 mM *p*-nitro blue tetrazolium chloride, 0.5 mM 5-bromo-4-chloro-3-indoylphosphate *p*-toluidine salt). After approximately 15 min development period, the color development reaction was stopped by placing the membrane in 5% acetic acid solution.

### Gel filtration chromatography

The partially purified TP by DEAE-cellulose ion exchange chromatography was analyzed on a Sephadex G-150 column (2.6 x 60 cm) and the hydroxyapatite homogeneously purified TP on a Sephadex G-100 column (1.2 x 60 cm). In both cases the columns were pre-equilibrated with Buffer A and calibrated with standard proteins: ovalbumin (*M*_r_, 43 kDa), aldolase (*M*_r_, 158 kDa), catalase (*M*_r_, 232 kDa), and ferritin (*M*_r_, 440 kDa). The columns were eluted with 400 mL of Buffer A. Fractions of 6.5 mL were collected at 4°C and 30 ml/hr flow rate. Eluted proteins were monitored by absorbance at 254 nm. Assay for TP activity was determined in all fractions.

### Chromatofocusing chromatography

Partially purified TP from the Sephadex G-150 gel filtration chromatographies was used for pI determination. A column (2.6x16 cm) was packed with Polybuffer Exchanger 94 pre-equilibrated with starting buffer (25 mM Imidazole-Cl, pH 6.8) buffer. 70 mL of the partially purified TP (35 mg) were applied to the column The column was first washed with 400 mL of the starting buffer, then eluted with 800 mL of a diluted (1:8, v:v) Polybuffer 74-Cl (pH 4.0) and 12 mL fractions were collected at flow rate of 30 mL/hr. pH was continuously monitored by an on-line Fisher Model 609 digital pH meter (Fisher Scientific, Pittsburgh, PA).

### Sedimentation equilibrium analysis

Sedimentation equilibrium was measured in a Beckman Instruments XL-A analytical ultracentrifuge using an AN60Ti rotor at 15,000 rpm at 20 ^o^C. The partial specific volume of TP (0.738) and sequence molecular weight (49,991 Da) were calculated using the UltraScan data analysis software package (Beckman Instruments, Inc., Palo Alto, CA). Three standard double sector cells were loaded with 150 μL solution containing different concentrations of the purified enzyme in Buffer A. The reference sector contained buffer A. Absorbance scans of TP as a function of radial position were made at 278 nm. The time required to attain equilibrium was established by subtraction of data sets until there was no observable difference greater than 0.01 absorbance units with no systematic deviation. Equilibrium was achieved in 48 hr. The analysis of the sedimentation equilibrium data followed a modeling strategy as previously described [31]. Software programs of the data analysis package of the XL-A analytical ultracentrifuge (Beckman Instruments, Inc., Palo Alto, CA) were used to model the sedimentation equilibrium data for TP. Global non-linear regression analyses of the total concentration as a function of radial distance for all three concentrations were performed to determine the monomer molecular weight, association constants, and stoichiometries for the selected association model which best fits the data [32]. For a self-associating system, monomer to n-mer at sedimentation equilibrium, the total solute concentration versus the radial position is the sum of the exponential distributions of monomer and n-mer species. Since the concentration of each n-mer is related to the monomer concentration (C_1_) by the mass action law (C_n_) = *K*_n_(C_1_)^n^, an exponential model for the total concentration as a function containing the following fitting parameters: monomer concentration at a selected radial reference position; each association constant *K*_n_ for the formation of n-mer from monomer; M_1_ the monomer molecular weight and; a base line offset between the reference and sample solution can be derived. The criteria for goodness of fit are that the error of optical detection be small (usually 0.01 AU) and random, *i*.*e*., has a small systematic error in the residuals. In addition, a correct monomeric molecular weight should be obtained by the fit [33].

### Mass spectrometry

The homogeneously purified TP (30 μg protein), extensively dialyzed in water and lyophilized and dissolved in 50% acetonitrile containing 0.1% formic acid, was injected into a Perkin Elmer SCIEX API III Triple Quadrupole Mass Analyzer (Perkin Elmer Inc., Norwalk, CA) by electrospray mode. Mass spectral analysis was also performed with a Matrix Assisted Laser Desorption Ionization Time of Flight (MALDI-TOF) Mass Spectrometer (Perceptive Biosystems Inc., Framingham, MA). Approximately 1 μg of the purified TP was mixed with a saturated solution of sinapinic acid, containing 10 μg of BSA (used as an internal standard). The sample was loaded into a sample plate and analyzed in the mass analyzer.

### *N*-terminal amino acid sequencing analysis

For *N*-terminal amino acid sequencing analysis, 10 μg of the homogeneous TP was directly applied into the reaction chamber in a Hewlett Packard Protein Sequencer Model G1005A (Hewlett-Packard Company, San Fernando CA) for 3 cycles. However, sequencing could not be accomplished because of the presence of an unknown blocking moiety on the *N*-terminal end of the TP resistant to Edman degradation. Deblocking of *N***-**formyl group and *N*-terminal pyrrolidone carboxylic acid in the proteins by anhydrous hydrazine [34] was unsuccessful.

### *CNBr* cleavage of the methionyl-X bond

Homogeneous TP (160 μg protein) was dissolved in 2 mL of Met-X cleavage reaction mixture (70% formic acid, 15 mM CNBr). High quality nitrogen gas was layered on top of the reaction mixture and incubated in the dark at room temperature for 15 hr. Fifteen mL of d.d water was added to the Met-X peptide bond digestion reaction mixture. The proteins were then lyophilized and dissolved in 5 mL of d.d water three times. Finally, the powder of digested mixture was dissolved in 50 μL of d.d water and stored at -70°C until it was used.

Tricine-SDS-PAGE [35,36] was performed using Hoeffer SE 600 electrophoresis apparatus (Hoeffer Scientific, Pharmacia, Piscataway, NJ). The gel dimension was 15 x 15 x 0.1cm and consisted of three layers of different gels; a 9-cm separating gel (5.3% acrylamide), a 3.5- cm spacer gel (1.92% acrylamide), and a 2.5-cm stacking gel (5% acrylamide). The purified TP treated with CNBr was dissolved directly in the loading buffer (125 mM Tris-Cl, pH 6.8; 2.9 M glycerol, 4% SDS, 0.06% bromophenol blue) and incubated in a boiling water bath for 3 min. The sample was then loaded and electrophoresis was performed at 28 mA constant current for about 15 hr until the tracking dye approached the bottom. The separated peptides were transferred to an Immobilon-P membrane as described above for the immunoblotting electrotransfer procedure. After the transfer, the membrane was stained with Coomassie Brilliant blue R-250, destained, rinsed in water and dried. Stained bands were cut out and the membrane slips were loaded directly into the automatic protein sequencer one by one.

### Aspartyl-prolinyl (D-P) peptide bond cleavage

Pure TP (100 μg) was dissolved in 100 μL of 75% formic acid and incubated for 20 hr at 37°C [37]. The reaction was terminated by the addition of 2 mL of d.d. water and the digested peptides mixture was lyophilized and then dissolved in 2 mL of d.d. water three times. The powder of digested peptides mixture was then dissolved in 50 μL of d.d. water and the mixture subjected to the Tricine-SDS-PAGE and immunoblotting as described above.

## Results and discussion

### Enzyme purification

This is the first report on purification of TP from human liver. Human hepatic TP was purified 5000-fold, to apparent homogeneity and a specific activity of 18,480 :mol/min/mg protein with a relatively high yield (40%) from large amounts of liver (200 g). The purification was accomplished using three column chromatographies; DEAE-cellulose anion exchange, Phenyl Sepharose CL-4B hydrophobic interaction, and hydroxyapatite chromatography. Table 1 shows the summary of the purification results. The present purification procedure proved shorter and more efficient to purify TP from human tissues on a large scale using larger amount than the previously reported methods [38–40].

**Table 1:** Purification of TP from human liver.

Purification procedures	Volume (mL)	Protein Concentration (mg protein/mL)	Total Protein (mg protein)	Specific activity (μmol/min/mg protein)	Total Activity (μmol/min)	Purification fold	Yield (%)
50,000 x g cytosol	600	40	24,000	3.6	87	1	100
DEAE-cellulose	1,800	3	5,400	16	85	4	98
Phenyl Sepharose CL-4B	625	0.25	156	472	74	130	85
Hydroxyapatite 2nd peak	5	2.7	13.5	68	0.9	19	1
Hydroxyapatite 1st peak	5	0.38	1.9	18,480	35	5,130	40

**Fig 1:**
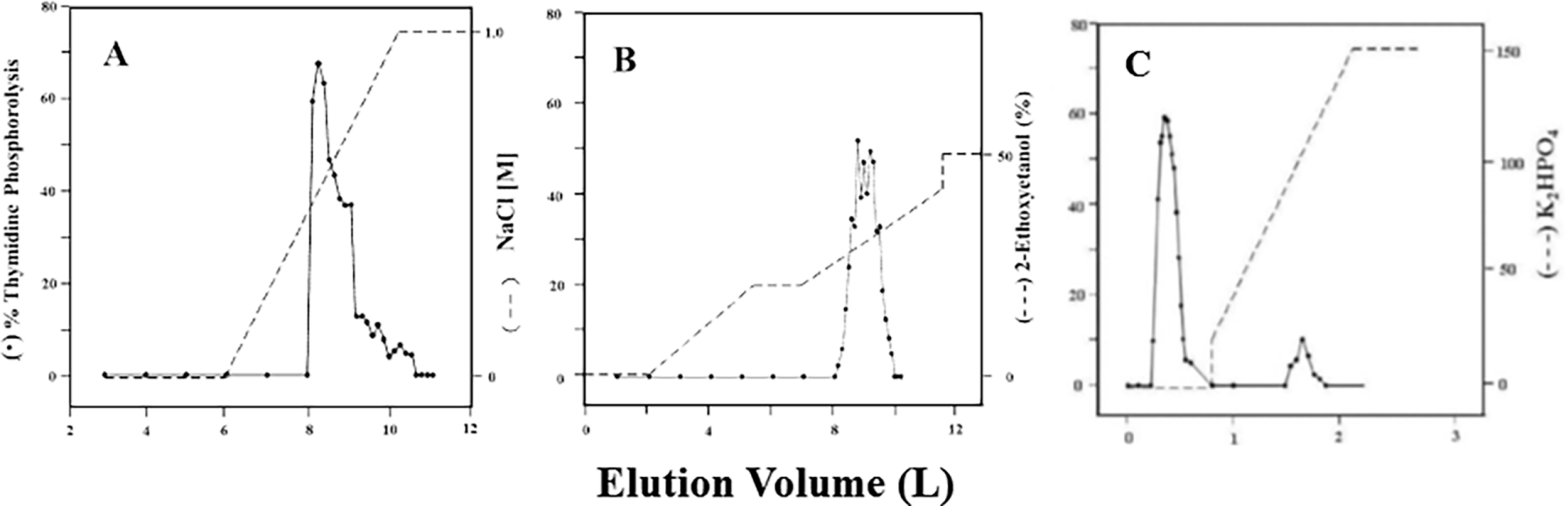
Purification of human TP by column chromatography. (**A**) DEAE-cellulose ion exchange chromatography, (**B**) Phenyl Sepharose CL-4B chromatography, (**C**) Bio-Gel HTP Hydroxyapatite chromatography.

**Fig 2:**
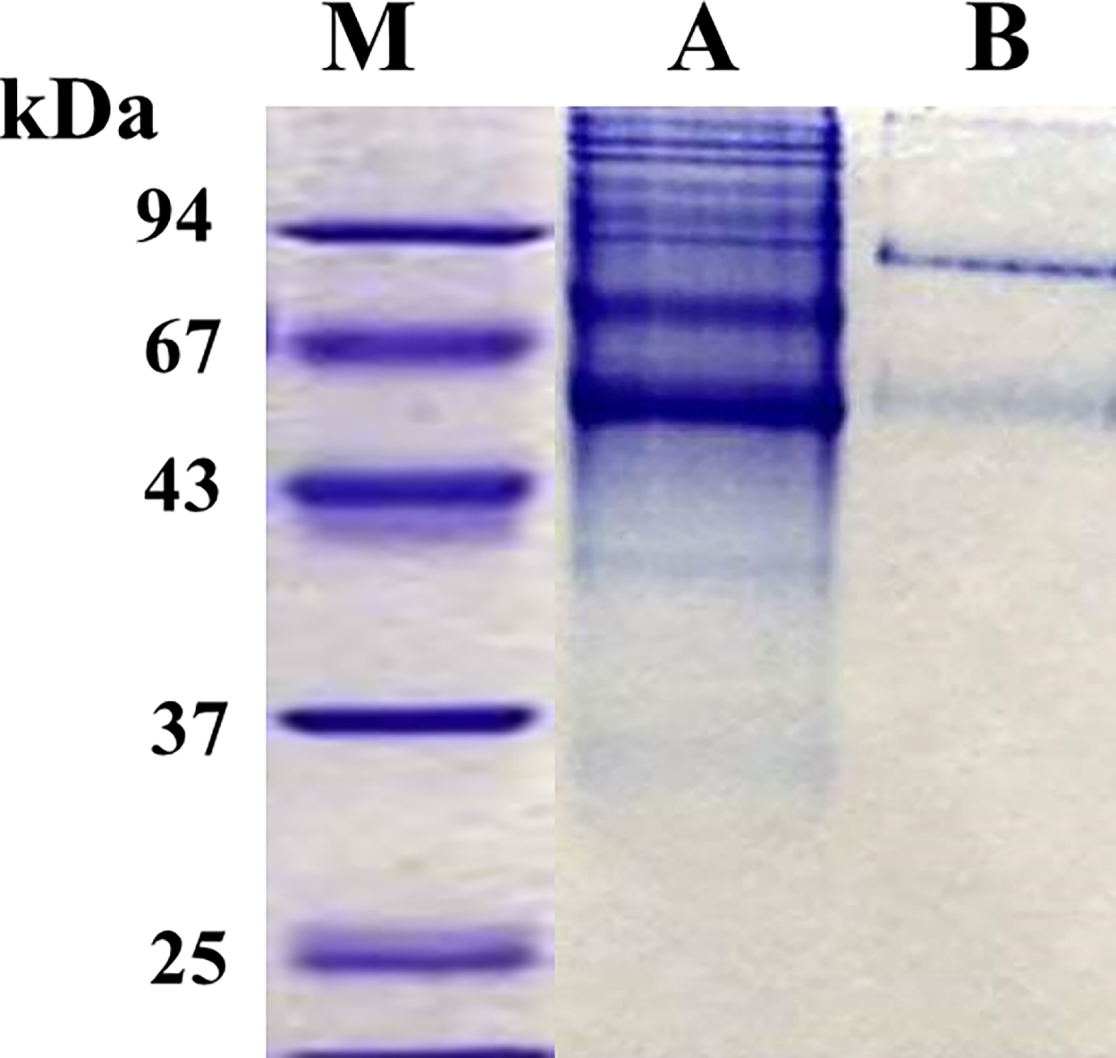
Native-PAGE of human hepatic TP eluted from the hydroxyapatite column. *Lane* ***M***, Molecular weight markers, *Lane* ***A***, Second peak (50 μg protein), *Lane* ***B***, First peak (5 μg protein) stained with Coomassie blue.

**Fig 3:**
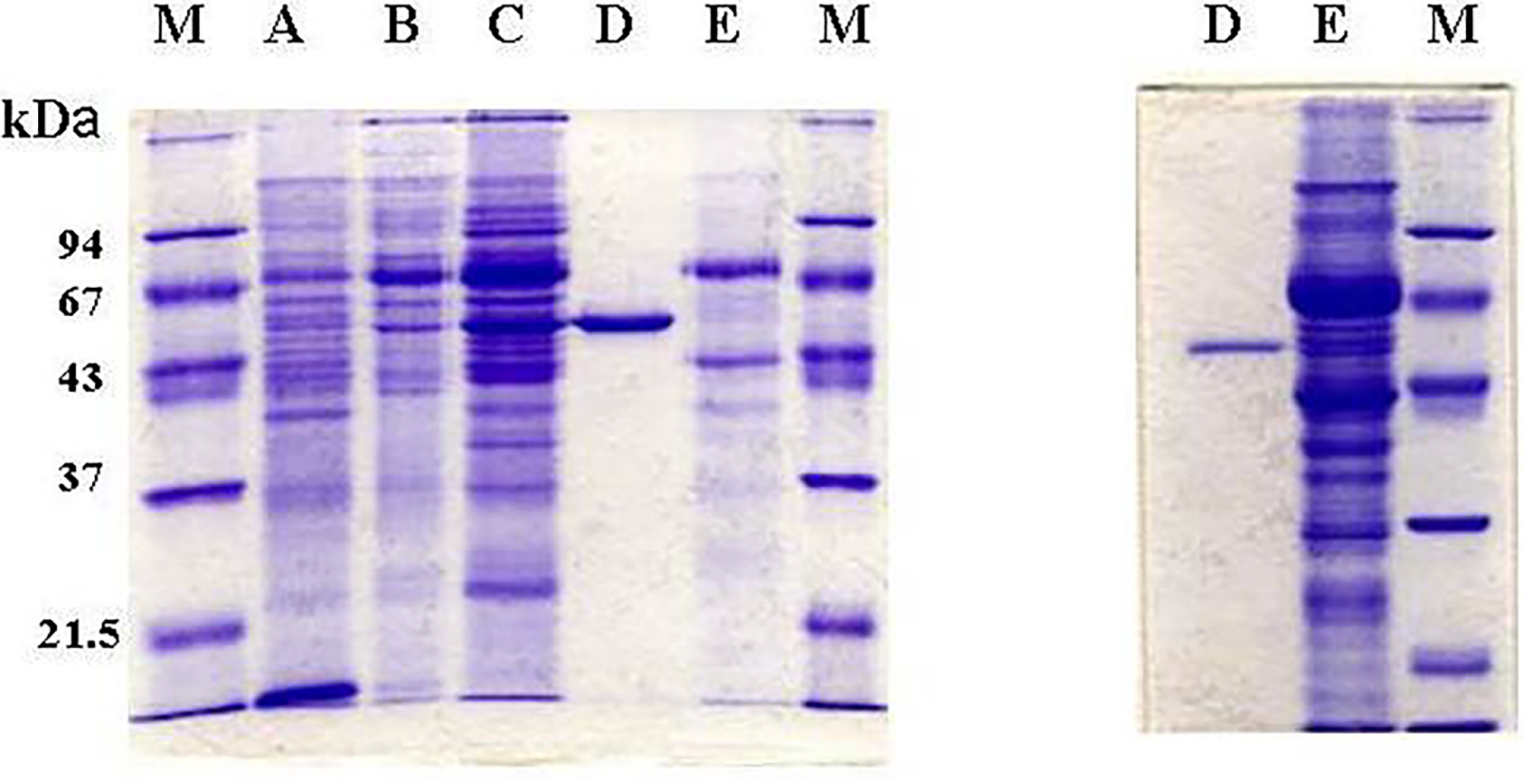
SDS-PAGE analysis for homogeneity and relative molecular weight of human liver TP isolated by column chromatography. Left gel was stained by Coomassie blue and the right gel by silver staining technique. *Lane* ***A***, cytosolic extract of human liver (15 μg protein); *Lane* ***B***, DEAE- cellulose fraction (15 μg protein); *Lane* ***C***, Phenyl Sepharose CL 4B fraction (20 μg protein); *Lane* ***D***. Hydroxyapatite first peak fraction (10 μg protein); *Lane* ***E***, Hydroxyapatite second peak fraction (10 μg protein); *Lane* ***M***, Molecular weight markers.

Fig 1**A** shows that TP bound to DEAE-cellulose ion exchange column and eluted with NaCl (0–1.0 M) gradient as a sharp peak of activity. This step resulted in 4-fold purification of TP with a specific activity of 16 μmol/min/mg protein. TP also bound to Phenyl Sepharose CL-4B but eluted as multiple peaks of activity at about 25% ethylene glycol monoethyl ether (Fig 1**B**). This step resulted in 130-fold purification and a specific activity of 472 μmol/min/mg protein. Two peaks of TP activities eluted from the hydroxyapatite column (Fig 1**C**). A major peak of activity appeared first during the wash with 2 mM phosphate and a second minor peak eluted at about 100 mM phosphate during the application of the gradient. The first peak was purified more than 5000-fold to a specific activity of 18,480 μmol/min/mg protein with 40% yield. The second peak was purified 19-fold to a specific activity of 68 μmol/min/mg protein with a yield of 1%. When the second peak was dialyzed to 1 mM phosphate and re-chromatographed on the hydroxyapatite column, it eluted as one peak in the wash by 2 mM phosphate as was the case with the first peak (data not shown). Thus, it appears that TP in the second peak was confounded with impurities. Indeed, native-PAGE (Fig 2) and SDS-PAGE (Fig 3) showed that the second peak appeared as multiple bands proving its impurity. On the other hand, the first peak appeared as one band, corresponded to approximately 100kDa (Fig 2), without any discernible bands of impurities. The stain density also increased as the amount of loaded protein was increased (data not shown). These results demonstrate the homogeneity of the first peak TP.

The elution of the major TP activity at a low concentration of 2 mM potassium phosphate indicates that human liver TP interacts very weakly or does not bind at all to the inorganic crystalline matrix resin of hydroxyapatite. This contrasts with the enzyme from other human tissues (platelet, etc.) which showed some interaction with hydroxyapatite resin and were eluted at higher concentration of phosphate (≥ 20 mM) [37–39]. This difference in the binding and elution pattern of human liver TP by hydroxyapatite column chromatography, gives additional credence to the notion that human liver TP is different from the enzymes from other human tissues. This proposition is further advanced by the results of molecular weight, immunoblot, and sedimentation equilibrium analyses as described below.

### Molecular weight

**Fig 4:**
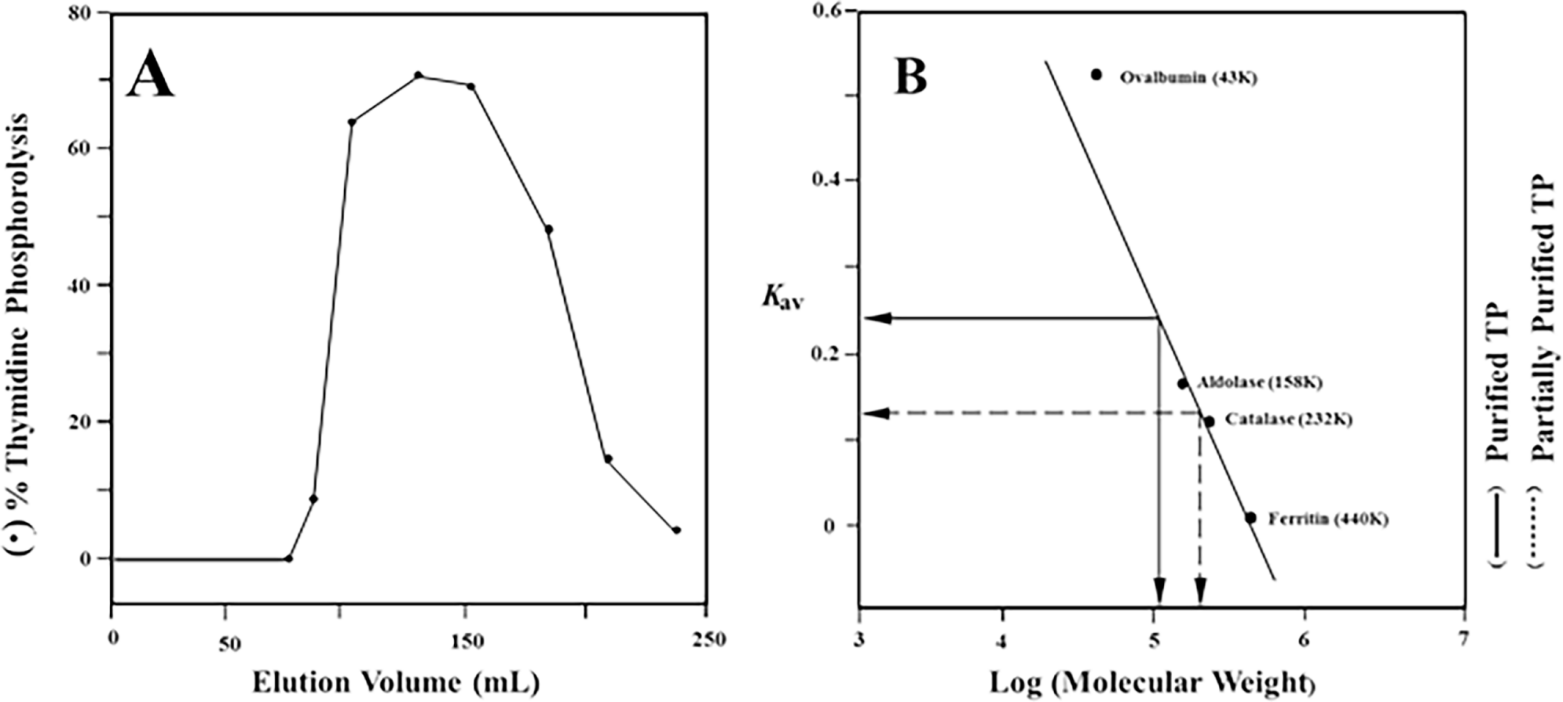
Molecular weight determination of human hepatic TP by gel filtration chromatography. (**A**) Chromatography of the DEAE-cellulose TP fraction on Sephadex G-150 column (**B**) Estimation of the native molecular weight of hydroxyapatite homogeneously purified enzyme (straight line) and the DEAE-cellulose partially purified TP (dashed line) by the use of chromatography on Sephadex G-100 column.

**Fig 5:**
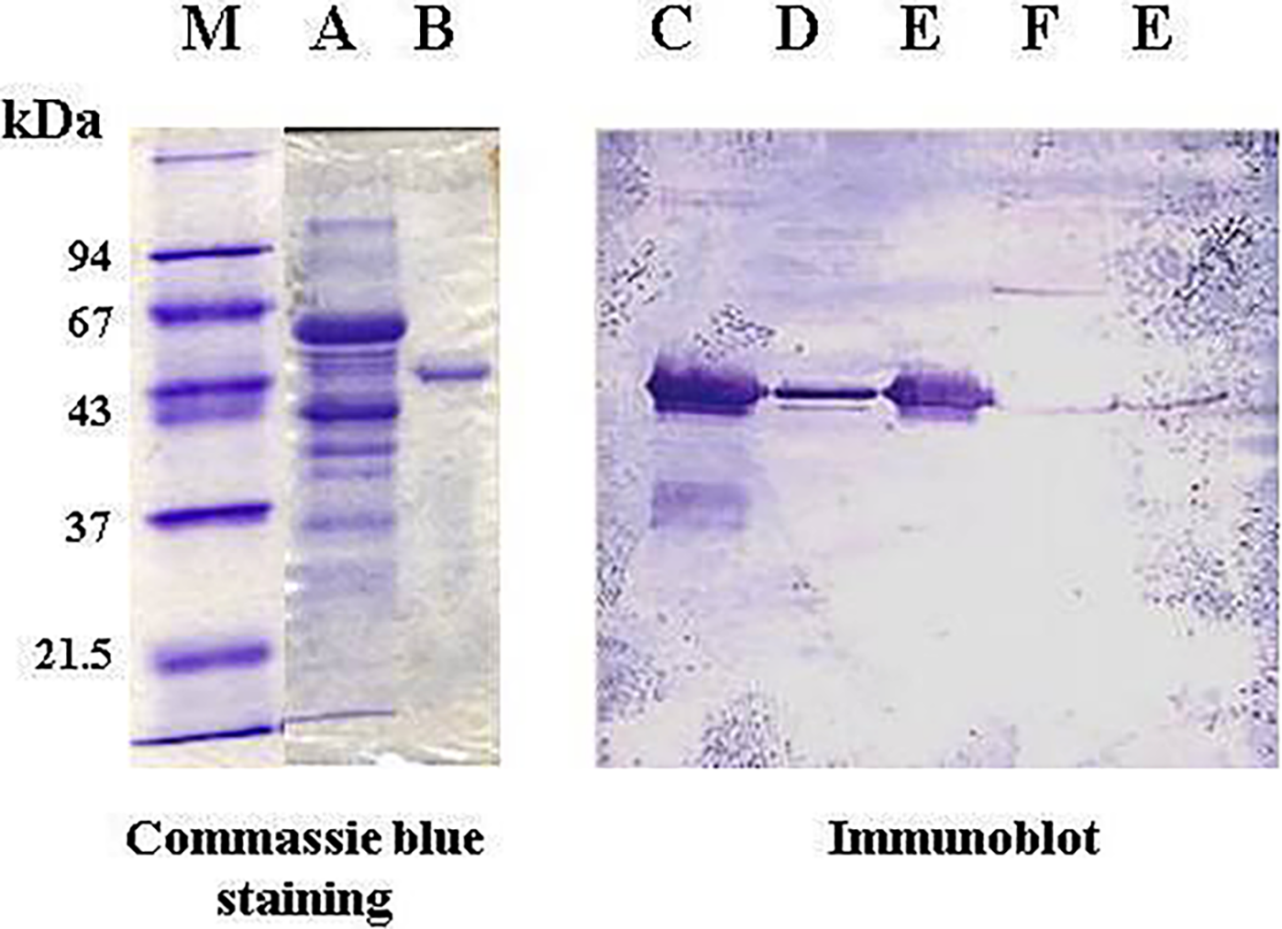
Immunoblot analysis using human placenta TP antisera binding to various preparations of hepatic TP after SDS-PAGE. Left gel; SDS-PAGE stained by Coomassie blue. Right gel; SDS-PAGE immunoblot stained with alkaline phosphatase. (**A**) Hydroxyapatite second peak fraction (50 μg protein). (**B**) Hydroxyapatite first peak fraction (5 μg protein). (**C**) Hydroxyapatite second peak fraction (50 μg protein). (**D**) Hydroxyapatite first peak fraction (5 μg protein). (**E**) Cytosolic extract of human liver (200 μg protein); (**F**) Cytosolic extract of human placenta (100 μg protein). (M) Molecular weight markers.

The relative molecular weight of the homogenous TP resulted from hydroxyapatite chromatography was approximately 100 kDa as estimated by Sephadex G-100 gel filtration (Fig 4B) and native-PAGE analysis (Fig 2). On the other hand, the molecular weight of the denatured enzyme was approximately 52 kDa as assessed by SDS-PAGE (Fig 3). This was further confirmed by immunoblotting. Fig 5 shows that antisera raised against human placenta TP bound to denatured hydroxyapatite two peaks as one major band of an identical molecular weight of 52 kDa. Therefore, it appears that human hepatic TP exists as a homodimer consisting of two identical subunits of 50 kDa each. The molecular weight of the subunit was further estimated by sedimentation equilibrium analysis as 49,885 kDa. Almost identical molecular weight of the subunit was obtained by two different types of mass spectrometry; 49,882 ± 1.2 by API III Triple Quadrupole Mass Analyzer, and 49,785 ± 5 daltons by MALDI-TOF Mass Analyzer.

The molecular weight and homodimer structure of human hepatic TP is similar to those of the enzymes from human amniochorion [38], blood platelet [39], placenta [40], psoriatic lesions [41] and the recombinant enzyme from a colorectal tumor [42]. Furthermore, the molecular weight of the human liver TP subunit is almost identical to the molecular weight of PD-ECGF (49,972 daltons) deduced from PD-ECGF cDNA sequence [43].

### Aggregation of human liver TP

Sephadex G-150 gel filtration of the native TP partially purified by DEAE-cellulose chromatography produced a broad peak of activity (Fig 4A) with average relative molecular weight of about 200 kDa (Fig 4B). This suggested that the enzyme in the DEAE-cellulose preparation may exist as an aggregate of 4 identical subunits. Therefore, probable molecular weight transition of human liver TP subunits was studied by native-PAGE, immunoblotting, and sedimentation equilibrium analyses.

**Fig 6:**
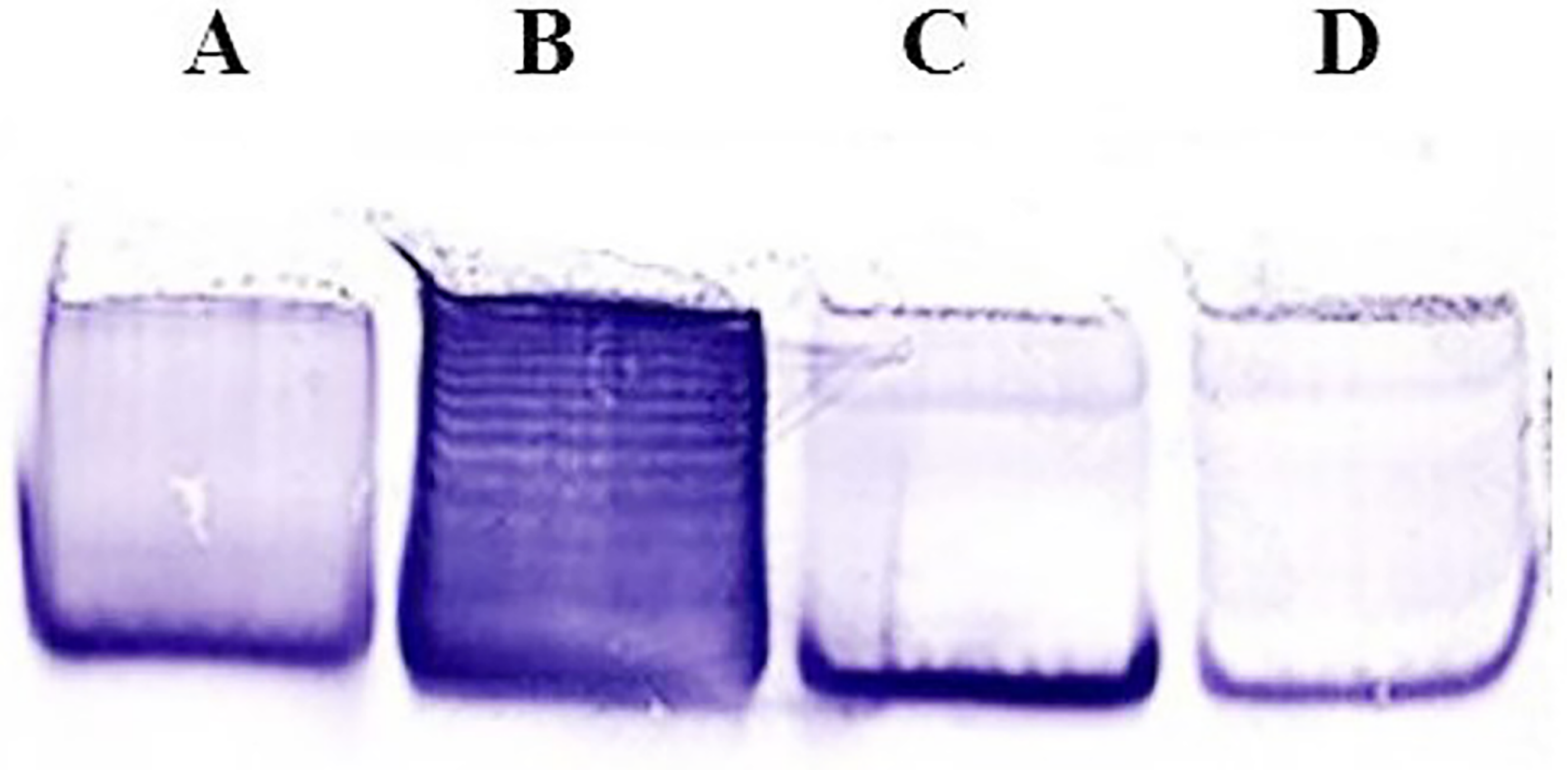
Immunoblot analysis using human placenta TP antisera binding to various preparations of hepatic TP after native-PAGE. (**A**) Cytosolic extract of human placenta (100 μg protein). (**B**) Cytosolic extract of human liver (200 μg protein). (**C**) Hydroxyapatite first peak fraction (5 μg protein). (**D**) Hydroxyapatite second peak fraction (50 μg protein).

**Fig 7:**
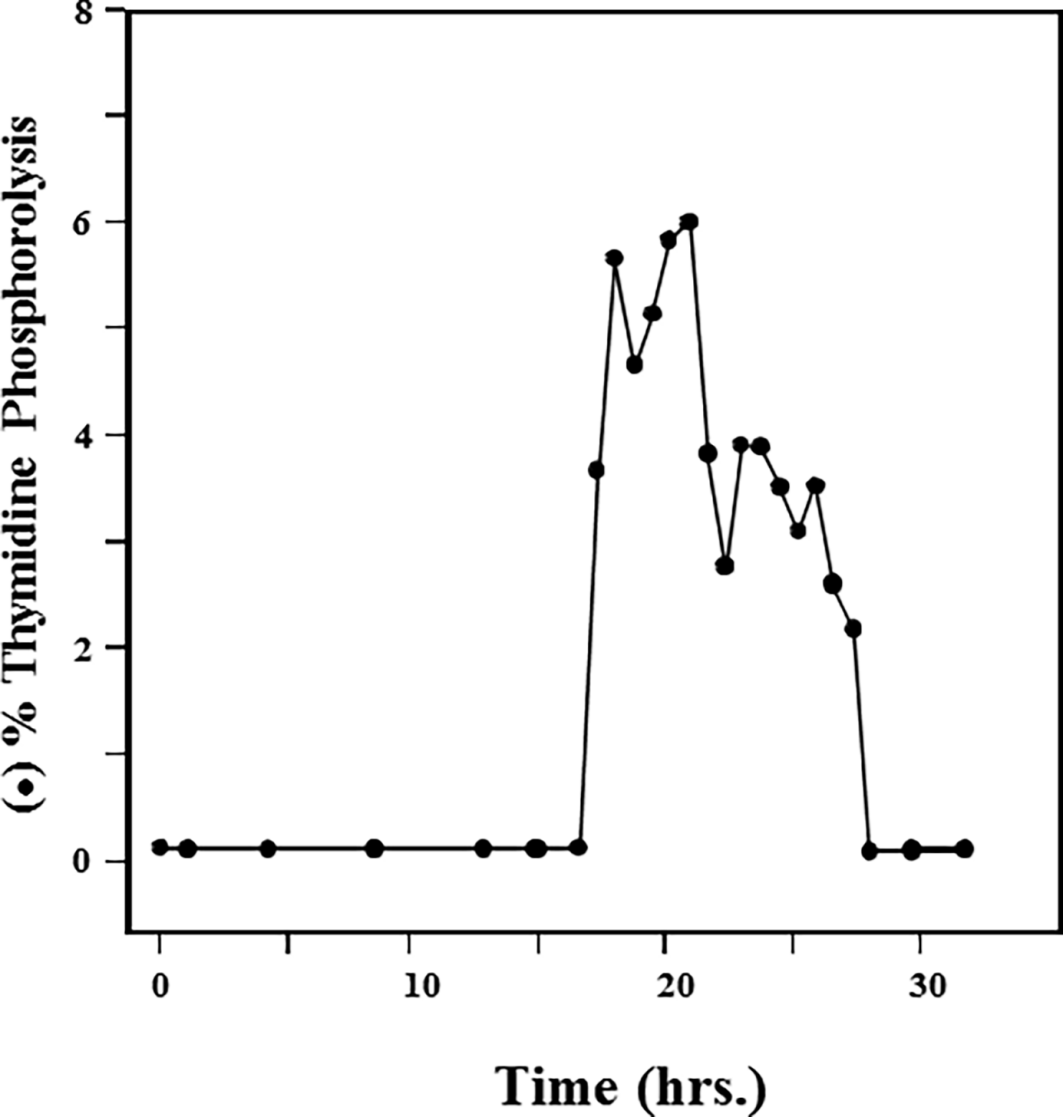
Preparative native-PAGE of TP in human liver cytosolic extract. Preparative native-PAGE was performed with an on-line electrophoresis system on a Bio-Rad 491 Prep Cell (Bio-Rad Laboratories, Hercules, CA) as described under Materials and Methods.

**Fig 8:**
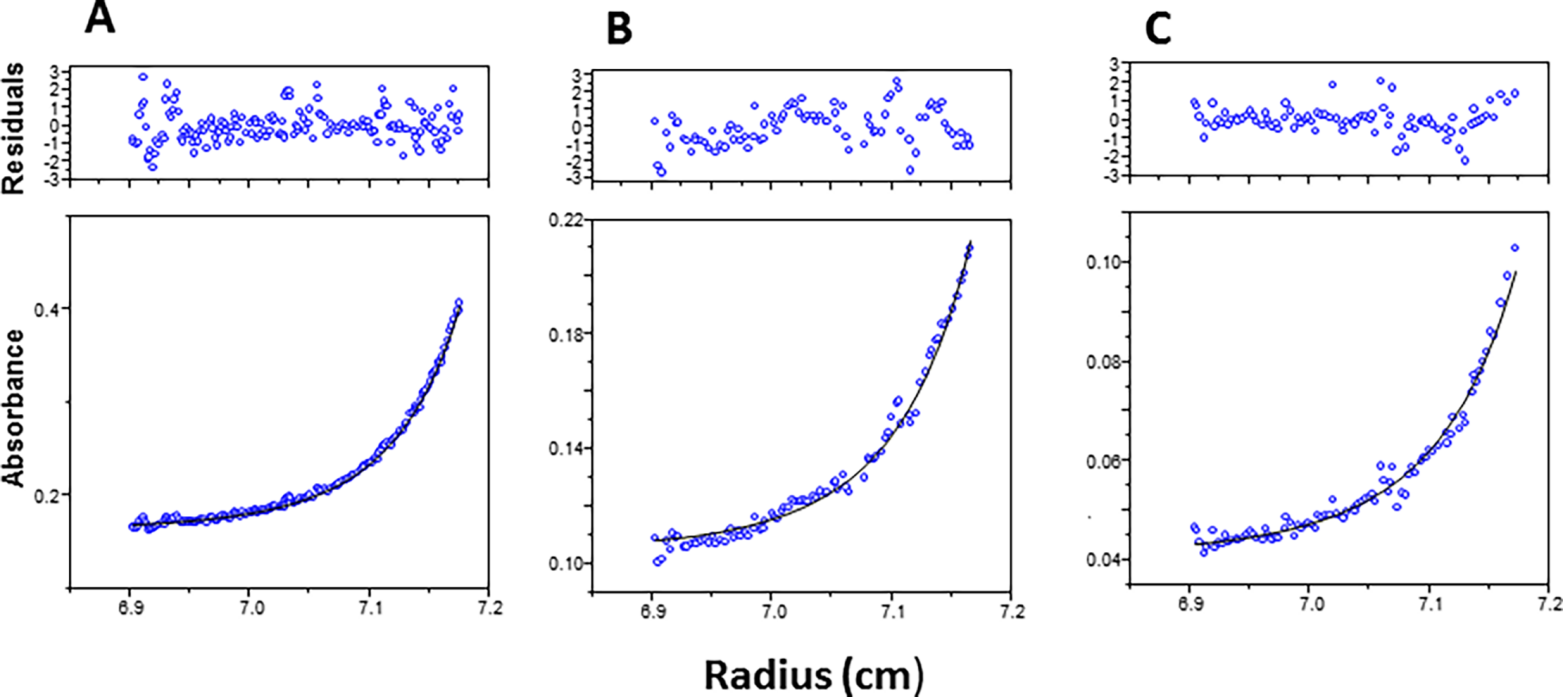
Sedimentation equilibrium analysis of human hepatic TP. The analysis of the sedimentation equilibrium data was performed as described under “Materials and Methods”. **A**, 0.4; **B**, 0.2; **C**, 0.1 mg protein/mL. **Top panel:** The weighted residuals (absorbance residual for each absorbance data point divided by the standard deviation of measurement for the data point) are plotted as a function of radial position. **Bottom panel:** Sedimentation equilibrium absorbance distribution of TP versus radial distance at 15,000 rpm and 20°C. Solid line through the open circle data points is the non-linear weighted regression fit using a monomer-dimer-tetramer association model. The global variables and 95% confidence limits that were obtained from this fit are given in Table 2.

Immunoblotting of various TP preparations with TP antisera were studied using human placenta cytosol as a positive control. Fig 6**C** and 6**D** show that both the homogeneous hydroxyapatite first and second peaks of TP activities have almost identical migration pattern. The human placenta cytosolic TP had a very similar migration pattern (Fig 6**A**). In sharp contrast, TP in human liver cytosol appeared as multiple bands with a clear ladder-like migration pattern (Fig 6**B**). Furthermore, a relatively broad elution profile of multiple major peaks of enzyme activities resulted from the preparative electrophoresis (Fig 7). These results suggest that TP in the liver cytosol exists not only as a dimer but also as larger aggregates. Indeed, sedimentation equilibrium analysis by analytical ultracentrifugation demonstrated that although the equilibrium association constant (*K*_a_) favors the formation of a 100 kDa dimer (Fig 8 and Table 2), TP can shift from a dimer to active tetramer, hexamer or bigger polymer. Different types of some aggregation states with unknown aggregation factor(s) could not also be excluded. In this regard, it is important to stress the point that TP in all preparations, whether crude or homogenous, is resolved by SDS to one monomer of approximately 52 kDa (Fig 5) further demonstrating the aggregated nature of hepatic TP.

**Table 2:** Equilibrium association constant (*K*_a_) of the purified TP in the non-linear regression fitting.

Model : Monomer—Dimer—Tetramer	
*K*_a (1,2)_ (M^-1^)	5.81 x 10^5^ (2.42 x 10^5^, 6.85 x 10^5^)
*K*_a (2,4)_ (M^-1^)	1.01 x 10^4^ (4.32 x 10^2^, 6.92 x 10^4^)
Monomer m.wt.	49,885.5 (48,890.7, 55,224.8)

For the transition model of the subunits aggregation, the non-linear regression fitting yielded the equilibrium association constants (*K*_a_) in absorbance units. For a monomer-dimer association the relationship for obtaining the molar association constant was *K*_molar_ = *K*abs x 0.5 (*E*_molar_). The path length of the cell was 12 mm. A *K*_a_ of 40.8 was obtained from the fit and converted to the molar terms using a molar extinction coefficient of 23740, which was calculated from the amino acid composition of PD-ECGF with 4 tryptophans, 1 tyrosine and 2 cystines [44].

It should be mentioned that a long storage of dimeric human amniochorion TP resulted in loss of activity and the formation of larger aggregates (e.g. trimer, tetramer etc.) of the enzyme [38]. This does not resemble the situation with hepatic TP where aggregation was observed in fresh preparations. Furthermore, this type of aggregation seems to be peculiar to hepatic TP since the placenta enzyme did not exhibit such aggregation pattern under the same conditions (Fig 6A). Whether or not hepatic TP aggregation process have a specific or nonspecific mechanism with any significant physiological role remains to be elucidated.

### Isoelectric point

Human liver TP activity has an isoelectric point 4.3 as determined by chromatofocusing chromatography. The pI value of 4.3 resembles those values determined for TP from human leukocytes [2,45], platelets [39], placenta [40] and psoriatic lesion [41] but lower than that reported for mouse liver TP [25, 45]. The low isoelectric point of the human liver TP could explain its strong binding to the DEAE-cellulose anion exchanger resin at pH 8.0.

### *N*-terminal amino acid sequencing analysis

The purified TP was blocked in the *N*-terminal amino acid moiety. The blockage could not be removed by Edman degradation method for amino acid sequence or by anhydrous hydrazine vapor analysis of the protein, indicating that the modification of the *N*-terminal amino acid of TP is neither *N*-formyl nor *N*-pyrrolidone carboxylic acid moieties. The inability to deblock the modified *N*-terminal amino prevented the sequencing of the *N*-terminus of the human liver TP.

### Amino acid sequence homology between hepatic TP and PD-ECGF.

**Fig 9:**
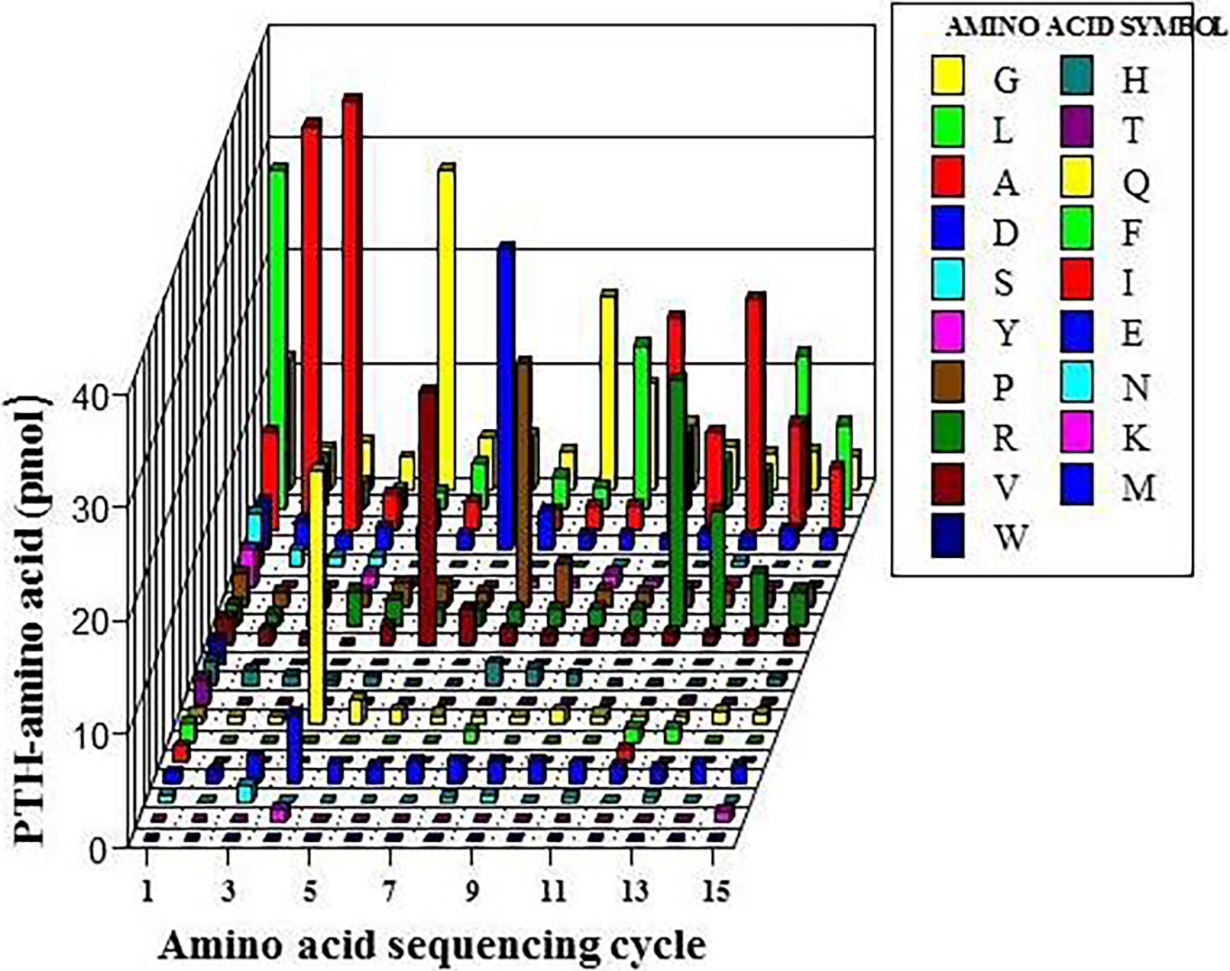
Amino acid sequence of the initial 15 residues (LAAQVDPGLARALX) of the 15 kDa peptide after the homogeneously purified TP from human liver was cleaved by CNBr. X-axis represents the phenylthiohydantoin (PTH) derivative amino acid followed by the Edman degradation in an automatic amino acid sequence analyzer (G1005AHewelett Packard protein sequencer). Y-axis represents the sequencing cycle in the analyzer.

Partial amino acid sequence analysis was performed to detect possible amino acid sequence homology between hepatic TP and PD-ECGF. Chemical cleavage by CNBr, acting on methionyl-amino acid peptide bond, resulted in three different digested peptides; 19 kDa, 15 kDa and 6 kDa as detected by Tricine-SDS-PAGE. The digested 15 kDa peptide was subjected to amino acid sequencing analysis for 30 cycles A 31 amino acid sequence of the *N*-terminal of the peptide was acquired with a high dependability and determined as M-LAAQGVDPGLARALXSGSPAERRQLLPRAR (Fig 9). The signal for the sixteenth residue (X) from the first methionine residue could not be recognized at the fifteenth cycle suggesting that the unidentified residue could be a cysteine. Signal for cysteine cannot be detected without methylation of the residue. Another 21 amino acid residues at the *N*-terminal of the19 kDa peptide were recognized as M-DGAGPPDLRDLVTTLGGALL, including the first methionine residue outside the amino acid sequencing procedure. This 51 amino acid sequence in human hepatic TP has 100% homology to the deduced amino acid sequence of PD-ECGF cDNA [43], indicating considerable homology between human liver TP and PD-ECGF.

Amino acid sequence analysis was further processed to detect possible additional amino acid sequence homology between hepatic TP and PD-ECGF. There are two aspartyl-proline (D-P) peptide bonds located at positions 28–29 and 353–354 in the amino acid sequence deduced from the PD-ECGF cDNA [43]. The amino acid sequence of the TP 15 kDa peptide has one D-P bond which is comparable to the D-P bond (353–354) of PD-ECGF. The possibility of another D-P bond in hepatic TP corresponding to the second D-P bond (28–29) of PD-ECGF [43] was examined by specific D-P peptide bond cleavage and amino acid sequence analysis. The enzyme was treated with formic acid (75%) at 37°C to break possible D-P bonds. Two peptides, 50 kDa and 52 kDa were discernible by Coomassie blue staining in Tricine-SDS-PAGE. The amino acid sequence of the *N*-terminal of the 50 kDa peptide was D-PSPEPKQ and included the aspartic acid residue cleaved by the D-P peptide bond digestion. These results verified the existence of a second D-P bond near the *N*-terminal of hepatic TP. Furthermore, the resulted 8 amino acid sequence was 100% homologous to residues 28–35 of the deduced PD-ECGF [43]. In addition, 6 amino acid residues; MTPGTG, were identified from sequencing the 52 kDa peptide. This sequence was also completely homologous to residues 5–10 of PD-ECGF [43].

In summary, a total 65 amino acids residues in human liver TP were derived from the sequence of the 30 amino acids (346 to 376 except 280-not detected) of the 15 kDa peptide; the 21 amino acids (294 to 314) of the 19 kDa peptide; the 8 amino acids (28 to 35) of the 50 kDa peptide; and the 6 amino acid (5 to 10) of the 52 kDa peptide. This 65 amino acid sequence in hepatic TP has 100% sequence and location homology to the deduced amino acid sequence of the PD-ECGF cDNA [43]. The identified M-L (15 kDa), M-D (19 kDa) and D-P (50 kDa) bonds were matched to those (346–347, 294–295, and 29–30, respectively) of the PD-ECGF amino acid sequence. These results further confirm that human liver TP, as the enzyme from other tissues, has great amino acid sequence homology with PD-ECGF. The results also demonstrated that the *N*-terminal of human liver TP is blocked contrary to what was reported for PD-ECGF [46]. The significance of the *N*-terminus blockage of hepatic TP in contrast to PD-ECGF remains to be elucidated. However, such blockage may have a potential physiological role. It was recently shown that PD-ECGF contributes to platelet activation and promotes thrombosis via its *N*-terminal SH3 domain binding proline-rich motif [47]. Therefore, the blockage or modifications of human liver TP *N*-terminus could suggest that the human hepatic enzyme has little or no role in thrombosis and is mainly concerned with pyrimidine salvage and metabolism. However, such postulate must wait for further evidence.

### Substrate specificity

Table 3 shows the substrate specificity of human liver TP with different natural and chemotherapeutic pyrimidine nucleosides. Human liver TP is highly specific to 2'-deoxyribosides with 2'-deoxyuridine being the best substrate. The enzyme is capable also of the phosphorolysis of 5'-deoxy-5-fluorouridine but to a lesser extent than the 2'-deoxyribosides. Uridine is a very poor substrate for the enzyme. However, the riboside, 5-fluorouridine, was relatively a good substrate. The activity of hepatic TP towards 5'-deoxy-5-fluorouridine, 5-fluorouridine, and uridine contrasts with the low or no activity of TP from normal extrahepatic tissues against these substrates [20,48].

**Table 3:** Apparent *K*_m_ values and specific activity of human hepatic TP with different natural and chemotherapeutic nucleosides.

Substrate	Apparent *K*_m_ [μM]	Specific activity (nmol/min/mg protein)	% of activity with thymidine
Thymidine	285 ± 55	5,550	100
Uridine	275 ± 77	114	2
2'-Deoxyuridine	735 ± 75	8,910	161
5-Fluoro-2'-deoxyuridine	325 ± 27	8,100	146
5-Fluorouridine	1,300 ± 67	1,500	27
5'-Deoxy-5-fluorouridine	200 ± 19	1,500	28

Apparent *K*_m_ values are weighed means from at least two experiments ± Standard error

The reaction mixture for the assay was similar to the standard assay except for the substrates, their concentrations and incubation time; 0.3 mM [2-^14^C]thymidine (2.7 Ci/mol); 0.6 mM [2-^14^C]uridine (2.8 Ci/mol); 0.8 mM [2-^14^C]deoxyuridine (1.4 Ci/mol); 0.35 mM [2-^14^C]5-fluoro-2´-deoxyuridine (3.2 Ci/mol); 1.3 mM [2-^14^C]5-fluorouridine (0.9 Ci/mol); or 0.2 mM [6-^14^H]5´-deoxy-5-fluorouridine (0.35 Ci/mol) and 20 μL of enzyme (76 ng protein), in a final volume of 40 μl. The concentrations for substrates were chosen at their apparent *K*_m_ except for thymidine and uridine were at their 2x *K*_m_s. The reactions were incubated for 5 min (except uridine and 5-fluorouridine for 30 min and 5´-deoxy-5-fluorouridine for 15 min) at 37°C. Under these conditions the enzyme activity was linear with time.

In conclusion, native human liver TP appears to be homologous to the enzyme from other tissues as well as the PD-ECGF in molecular weight, subunit structure and amino acid sequence. Nevertheless, the hepatic enzyme differs from other human TPs by its substrate specificity, weak interaction with hydroxyapatite resin and its existence in multimers. These multimer TPs are recognized by antibody binding (Fig 6) and are enzymatically active (Fig 7). The physiological reason and consequences of such differences are currently unknown but could be due to changes in conformation, hydrophobicity, etc. of the enzymes as a result of variations in the surrounding intracellular environment in the different tissues.

## Abbreviations

CNBr: Cyanogen bromide
PAGE: polyacrylamide gel electrophoresis
PD-ECGF: platelet derived-endothelial cell growth factor
SDS: Sodium dodecyl sulfate
TP: thymidine phosphorylase (EC 2.4.2.4)
TWEEN 20: polyoxyethylene (20) sorbitan monolaurate
